# Gene Expression of Leptin and Long Leptin Receptor Isoform in Endometriosis: A Case-Control Study

**DOI:** 10.1155/2013/879618

**Published:** 2013-03-24

**Authors:** Andrea Prestes Nácul, Sheila Bunecker Lecke, Maria Isabel Edelweiss, Débora Martinho Morsch, Poli Mara Spritzer

**Affiliations:** ^1^Gynecological Endocrinology Unit, Division of Endocrinology, Hospital de Clínicas de Porto Alegre (HCPA), Rua Ramiro Barcelos 2350, 90035-003 Porto Alegre, RS, Brazil; ^2^Human Reproduction Unit, Hospital Fêmina, Grupo Hospitalar Conceição (GHC), Rua Mostardeiro 17, 90430-001 Porto Alegre, RS, Brazil; ^3^Division of Pathology, HCPA, Rua Ramiro Barcelos 2350, 90035-003 Porto Alegre, RS, Brazil; ^4^Laboratory of Molecular Endocrinology, Department of Physiology, Universidade Federal do Rio Grande do Sul (UFRGS), Rua Ramiro Barcelos 2350, 90035-003 Porto Alegre, RS, Brazil

## Abstract

In this study, leptin/BMI ratio in serum and peritoneal fluid and gene expression of leptin and long form leptin receptor (OB-R_L_) were assessed in eutopic and ectopic endometria of women with endometriosis and controls. Increased serum leptin/BMI ratio was found in endometriosis patients. Leptin and OB-R_L_ gene expression was significantly higher in ectopic versus eutopic endometrium of patients and controls. A positive, significant correlation was observed between leptin and OB-R_L_ transcripts in ectopic endometria and also in eutopic endometria in endometriosis and control groups. A negative and significant correlation was found between OB-R_L_ mRNA expression and peritoneal fluid leptin/BMI ratio only in endometriosis. These data suggest that, through a modulatory interaction with its active receptor, leptin might play a role in the development of endometrial implants.

## 1. Introduction

Endometriosis is defined as the presence of endometrial glands and/or stroma outside the uterus. In women with endometriosis, the eutopic endometrium has specific characteristics that favor tissue survival, adhesion, and growth outside the uterine cavity. Several studies have demonstrated that endometriosis is associated with abnormal peritoneal and endometrial production of proinflammatory cytokines and growth and angiogenic factors [1, 2].

Leptin, a hormone produced mainly by adipocytes, is expressed in endometrium [3] and has been implicated in the regulation of sex hormone production, ovulation, endometrial cell physiology, and early embryo development and implantation [4]. It may also play a role in endometriosis through its inflammatory and angiogenic properties. Nevertheless, studies evaluating serum and peritoneal fluid (PF) levels of leptin in patients with endometriosis report conflicting results: some describe increased levels [2, 5–10], while others report no differences between patients with endometriosis and controls [7, 11–14]. Moreover, the possibility of an association between PF leptin levels and severity of endometriosis is also controversial, with some studies suggesting a negative correlation [2, 6, 8] and others showing a positive correlation with more severe forms of peritoneal endometriosis [5, 7, 13, 15]. 

Interestingly, only a few studies so far have evaluated leptin receptor gene and/or protein expression in endometrial tissue of women with endometriosis [16–18]. Lima-Couy et al. [16] evaluated the three isoforms of leptin receptor—total (OB-R_T_), long (OB-R_L_), and short (HuB219.3)—in the eutopic endometrium of patients with moderate and severe endometriosis. Those authors observed increased receptor expression in the period corresponding to embryo implantation, with no difference between patients and controls. Some authors [17, 19] have reported expression of leptin receptor in both eutopic and ectopic endometria.

Therefore, the aims of the present study were (a) to assess leptin and OB-R_L_ gene expression in ectopic and eutopic endometria of women with endometriosis and in eutopic endometrium of non-endometriosis controls, (b) to determine the leptin/BMI ratio in serum and PF in both groups, (c) to assess the immunoreactive presence of OB-R_L_ in endometrium and endometriotic implants, and (d) to investigate the relationship among these variables.

## 2. Materials and Methods

### 2.1. Subjects

The sample was selected among patients undergoing gynecological laparoscopy for infertility, pelvic pain, ovarian pathology, or tubal ligation (TL), between September 2007 and March 2009. Twenty-eight women with pelvic endometriosis and 17 women without laparoscopically proven endometriosis or other pelvic pathology were consecutively selected from this group. Infertility was defined as inability to achieve pregnancy after one year of unprotected sexual intercourse. Chronic pelvic pain was defined as noncyclical pelvic pain of sufficient severity to cause functional disability or lead to medical care, lasting six months or longer (American College of Obstetricians and Gynecologists). Endometriosis was confirmed by histology in all patients with suspected lesions at laparoscopy. Endometriosis was classified according to the revised classification of the American Society of Reproductive Medicine [20]. Peritoneal endometriotic lesions were observed in all patients, and superficial ovarian endometrioma was also found in two of them. 

Inclusion criteria were (i) premenopausal status, (ii) need for laparoscopy, and (iii) no use of hormonal medication in the previous three months. The sole exclusion criterion was body mass index (BMI) above 35. Participants presented neither metabolic comorbidities, such as diabetes, dyslipidemia, abnormal renal, or hepatic function, nor clinical evidence of systemic diseases or pelvic inflammatory disease. The study protocol was approved by the Research Ethics Committee at Hospital de Clinicas de Porto Alegre (IRB-equivalent), and written informed consent was obtained from all subjects.

### 2.2. Study Protocol

All participants underwent physical examination, including measurement of height and weight and estimation of BMI. Laparoscopy with biopsy of endometriotic implants and a concomitant biopsy of the eutopic endometrium were performed preferentially in the second half of the menstrual cycle. However, in about 20% of participants, laparoscopy and biopsy were performed in the proliferative phase. A single sample of superficial peritoneal endometriotic tissue was obtained from the largest lesion. Subcutaneous adipose tissue samples (approximately 1 cc) were also collected from the periumbilical region before the end of the procedure. Eutopic endometrial samples were collected using curettage. The same surgeon performed all laparoscopic evaluations (AN).

Peripheral venous blood samples were collected immediately before anesthetic induction for laparoscopy. PF samples were collected from the Douglas pouch immediately after the start of the procedure. All samples were kept on ice for transport to the laboratory and stored in aliquots at −80°C until assayed. Endometriotic and endometrial samples were fractioned: one portion was immediately frozen in liquid nitrogen and stored at −80°C until mRNA extraction, while the other was fixed in 10% buffered formalin and embedded in paraffin for subsequent histological diagnosis and immunohistochemistry, as previously described [21].

Based on histological and laparoscopic findings, three types of tissue were studied: (1) eutopic endometrium from nonendometriosis controls, (2) eutopic endometrium from patients with endometriosis, and (3) ectopic endometrium from patients with endometriosis.

### 2.3. Serum and Peritoneal Fluid Measurements

Serum estradiol and progesterone concentrations were assayed by electrochemiluminescence (Roche Diagnostic, Mannheim, Germany). Serum and peritoneal leptin levels were determined using a Human Leptin ELISA kit (LINCO Research, St. Charles, MO, USA). 

### 2.4. RNA Isolation

Endometrial and adipose tissue total RNA extraction was carried out in phenol/guanidine isothiocyanate (Trizol, Invitrogen Life Technologies, Foster City, CA, USA) as previously described [21, 22]. Concentration and quality of total RNA were assessed using a GeneQuant spectrophotometer (Pharmacia Biotech, Cambridge, England).

### 2.5. Real-Time Reverse Transcription-Polymerase Chain Reaction (RT-PCR) Protocol

Reverse transcription of 1 *μ*g of total RNA into cDNA was carried out using the Superscript II First-Strand Synthesis System for RT-PCR (Invitrogen Life Technologies, Foster City, CA, USA), according to the manufacturer's instructions, in a PCT-100 Programmable Thermal Controller (MJ Research Inc., Watham, MA, USA). 

Real-time PCR was performed in triplicate in a 7500 Fast real-time PCR System thermal cycler with 7500 Fast System Sequence Detection 1.4 Software (Applied Biosystems, Foster City, CA, USA). Experiments were performed by monitoring in real time the increase in fluorescence of the SYBR Green dye as previously described [23–25]. Primers were designed by Primer Express 3.0 Software for real-time PCR (Applied Biosystems, Foster City, CA, USA) and acquired from Invitrogen (Life Technologies, Foster City, CA, USA). Primer sequences were designed to target two exons of an mRNA sequence with respect to known splice variants and single-nucleotide polymorphism positions. The forward and reverse primer sequences designed for leptin (NM_000230.2) were (5′ to 3′) TCCCCTCTTGACCCATCTC and GGGAACCTTGTTCTGGTCAT, respectively. These primers anneal between residues 858 to 876 (forward) and 967 to 948 (reverse), producing a PCR product of 110 bp. The forward and reverse primer sequences for leptin receptor (NM_001003679.2) were (5′ to 3′) AGGAAGCCCGAAGTTGTGTT and TCTGGTCCCGTCAATCTGA, respectively. These primers anneal between residues 3,617 to 3,636 (forward) and 3,716 to 3,698 (reverse), resulting in an amplicon of 100 bp. Beta-2 microglobulin (NM_004048.2) was used to normalize mRNA quantitation. CTATCCAGCGTACTCCAAAG and ACAAGTCTGAATGCTCCACT (5′ to 3′) forward and reverse B2M primer sequences anneal between residues 119 to 138 (forward) and 283 to 264 (reverse), resulting in an amplicon of 165 bp. cDNA samples (1.0 ng/*μ*L) were mixed with a predetermined forward and reverse primer volume (0.9 and 0.7 *µ*L for leptin, 0.9 and 0.3 *µ*L for leptin receptor, and 0.7 and 0.9 *µ*L for B2M) and 12.5 *μ*L of 2X Fast SYBR Green Master Mix (Applied Biosystems, Foster City, CA, USA) in a total of 25 *μ*L. Protocol conditions consisted of denaturation at 94°C for two minutes followed by 50 cycles (30 s at 94°C and 30 s at 60°C). Primers generated amplicons that produced a single sharp peak during melting curve analysis. Data were analyzed by relative quantitation using the comparative C_T_ method [26]. Validation assays for endometrium and adipose tissue were performed by amplification of the target and reference genes, separately, using serial dilutions of an mRNA sample. Both target and reference mRNAs exhibited equal amplification efficiency. The ΔΔC_T_ method calculates changes in gene expression as relative fold difference between an experimental and calibrator sample, correcting nonideal amplification efficiencies [27]. 

### 2.6. Immunohistochemistry

Formalin-fixed, paraffin-embedded endometrial samples were cut into 5 *μ*m slices, which were stained by immunohistochemistry using the avidin-biotin-peroxidase method [28], as previously described [29, 30]. Following deparaffinization and rehydration with a graded series of ethanol, immunohistochemistry was performed using peroxidase reaction. Sections were incubated with 3% H_2_O_2_ at room temperature for five minutes in order to suppress endogenous peroxidase activity. The samples were then incubated in a humidity chamber at room temperature for 30 minutes with a 0.5 *μ*g/mL goat antihuman leptin receptor antibody C20 (Santa Cruz Biotechnology, Santa Cruz, CA, USA). Phosphate-buffered saline (PBS) containing 0.5% bovine serum albumin (w/v) replaced the primary antibody in negative controls. Samples of the choroid plexus were used as positive controls. After 20-minute incubation with the linker, streptavidin-peroxidase was used for five minutes to stain the slices. Subsequent to each incubation step, the tissues were washed three times with PBS 50 mM Tris–HCl buffer. Slices were counterstained with Mayer's hematoxylin and mounted. A positive reaction was characterized by the presence of granular brown staining in the cytoplasm. The intensity of immunostaining in epithelium and stroma was evaluated by two independent observers and classified as negative, weak, moderate, or intense and converted into arbitrary units on a semiquantitative scale of 0 to 3 [31].

### 2.7. Statistical Analysis

The sample size for detecting significant differences in serum leptin levels was estimated as 12 women with endometriosis and 12 controls, based on the study by Matarese et al. [2] and considering a power of 80% and alpha of 5%. Data are presented as mean ± SD or median and interquartile range. Comparisons between group means were analyzed by Student's *t*-test. Median values were compared using the Mann-Whitney *U *test. Comparisons of median values involving three groups were analyzed using the Kruskal-Wallis test. The chi-square test was used to compare qualitative variables. Pearson's rank or Spearman's correlation coefficients were calculated using a two-tailed significance test for variables with a Gaussian or non-Gaussian distribution, respectively. The nonparametric Wilcoxon signed-rank test was used if required based on the number of subjects and the nonhomogeneous features of each group. The gamma test was used for qualitative comparison of more than two groups.

All analyses were performed using the Statistical Package for the Social Sciences 16 (SPSS, Chicago, IL, USA). Data were considered significant at *P* < 0.05.

## 3. Results

Participants age ranged from 21 to 50 years. In one nonendometriosis control, endometrial biopsy was of insufficient quality for interpretation. Therefore, the results of gene expression and immunohistochemistry refer to data from 28 patients with endometriosis and 16 controls.

Endometriosis was classified as stage I (minimal) in 13 patients, stage III (moderate) in six, and stage IV (severe) in nine. Laparoscopy was performed for infertility investigation in 15 patients (33.3%), tubal ligation in 13 (28%), chronic pelvic pain in nine (20%), adnexal pathology in five (11%), association of infertility and chronic pelvic pain in two (4.5%), and association of tubal ligation and chronic pelvic pain in one (2.2%) patient.  Table 1 presents the clinical profile of the endometriosis and control groups. Circulating levels of estradiol and progesterone in the proliferative and secretory phases of the menstrual cycle in each group also appear in Table 1. While progesterone levels were higher in the secretory than in the proliferative phase, no differences were observed in estradiol and progesterone levels between the endometriosis and control groups. Serum leptin/BMI ratio was similar in the proliferative and secretory phases of the cycle in the endometriosis (0.56 (0.27–1.24) versus 0.61 (0.41–0.91) resp., *P* = 0.97) and control (0.33 (0.21–0.53) versus 0.37 (0.18–0.68) resp. *P* = 0.77) groups. PF leptin/BMI ratio was also found to be similar in the proliferative and secretory phases in the endometriosis (1.15 (0.35–1.97) versus 0.62 (0.39–1.04) resp. *P* = 0.64) and control (0.46 (0.12–0.72) versus 0.39 (0.26–0.74) resp., *P* = 0.73) groups. Therefore, posterior analyses were performed including all patients, without considering the cycle phase.

As shown in Table 2, the serum leptin/BMI ratio was significantly higher in the endometriosis group than in controls. A trend toward significantly higher PF leptin/BMI ratio was also observed in the endometriosis group (*P* = 0.07). There were no significant differences in serum and PF leptin/BMI ratio when controls were compared to patients with minimal/mild or moderate/severe endometriosis. 

**Figure 1:**
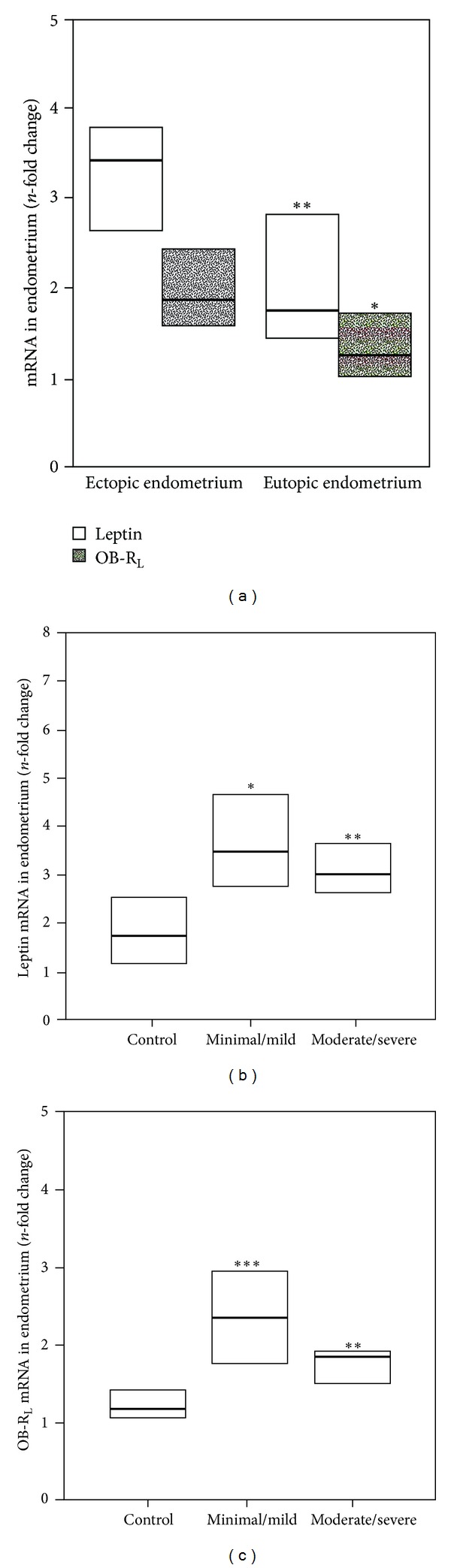
Leptin and long leptin receptor isoform (OB-R_L_) gene expression in endometriosis. (a) Ectopic endometrium and eutopic endometrium of patients with endometriosis. Values are expressed as *n*-fold difference in relation to the calibrator sample (ΔΔCt method). **P < 0.001 (leptin mRNA ectopic versus eutopic endometrium); *P < 0.05 (OB-R_L_ mRNA ectopic versus eutopic endometrium) (Wilcoxon signed-rank test). (b) Leptin gene expression and (c) long leptin receptor isoform (OB-R_L_) in eutopic endometrium of non-endometriosis controls and ectopic endometrium of minimal/mild and moderate/severe endometriosis. Values are expressed as n-fold difference in relation to the calibrator sample (ΔΔCt method). Asterisks indicate significant difference in comparison to controls. *P < 0.05; **P < 0.01 and ***P < 0.001(Mann-Whitney *U*).

Leptin mRNA and OB-R_L_ were detectable in all samples of ectopic endometrium. In the eutopic endometria of patients and controls, leptin mRNA and OB-R_L_ were detectable in 25 out of 28 and 16 out of 17 tested samples (89% and 94%, resp.).  Figure 1(a) shows that leptin mRNA expression was significantly higher in ectopic lesions than in the eutopic endometrium of patients with endometriosis (*P* < 0.001). OB-R_L_ mRNA expression was also significantly higher in ectopic lesions as compared to the eutopic endometrium of the endometriosis group (*P* < 0.05). 

Leptin (Figure 1(b)) and OB-R_L_ mRNA (Figure 1(c)) expressions were also significantly higher in ectopic endometrium when endometriosis patients were stratified into minimal/mild and moderate/severe endometriosis groups as compared to the eutopic endometrium of non-endometriosis controls. Conversely, no differences were found between endometriosis stages. Leptin and OB-R_L_ transcripts were also similar in the eutopic endometria of patients with endometriosis and controls (data not shown). In addition, no differences were found in leptin and OB-R_L_ gene expression in subcutaneous fat samples of nonendometriosis controls (leptin mRNA 1.95 (1.45–2.34) and OB-R_L_ mRNA 2.35 (2.02–2.57)) and samples of patients with endometriosis (2.21 (1.58–2.84) and 2.13 (1.98–2.67) resp.).

A positive and significant correlation was observed between leptin and OB-R_L_ transcripts in the ectopic endometrium of patients with endometriosis (*R* = 0.57,  *P* < 0.01) and in the eutopic endometrium of both endometriosis and control participants (endometriosis: *R* = 0.52,  *P* < 0.01; nonendometriosis controls: *R* = 0.57,  *P* < 0.02).

**Figure 2:**
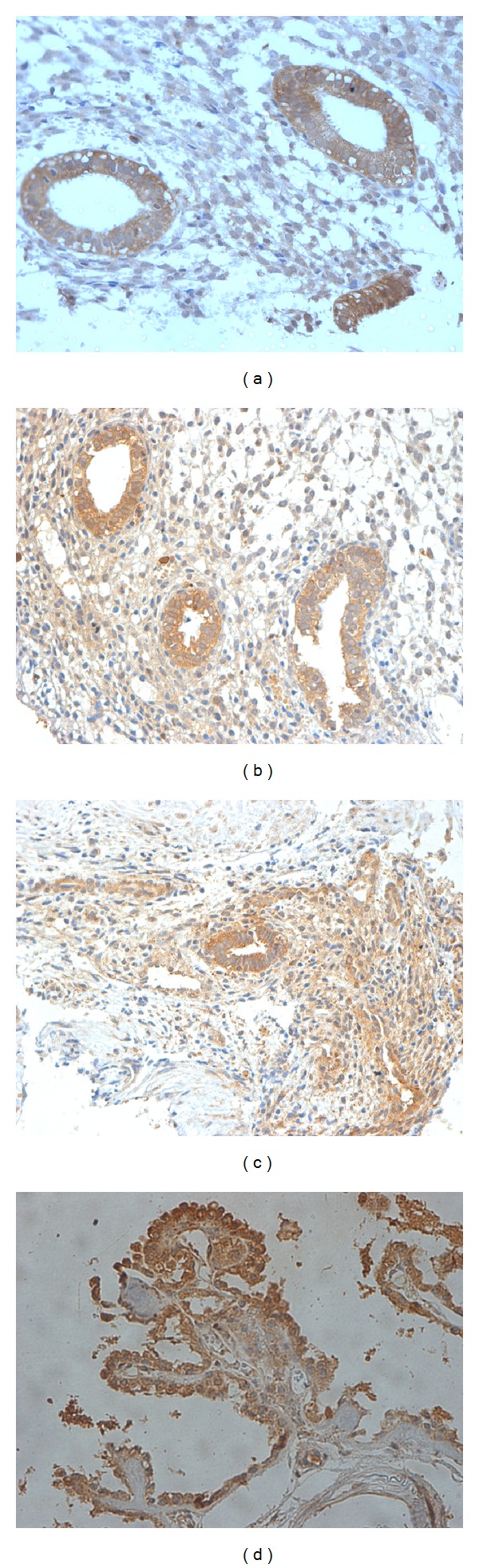
Immunostaining of long leptin receptor isoform (OB-R_L_) in eutopic endometrium. (a) Non-endometriosis controls, (b) eutopic and (c) ectopic endometria of patients with endometriosis, and (d) positive control (choroid plexus). Original magnification: 400x.

Figure 2 shows immunostaining of OB-R_L_ in representative biopsies of eutopic endometrium from the control and endometriosis groups in representative biopsies of ectopic endometrium and in positive control samples of the choroid plexus. Cytoplasmic staining was observed in both the stromal and epithelial compartments of women with different stages of endometriosis and controls. Intensity of OB-R_L_ immunostaining in moderate/severe endometriosis samples was similar to the OB-R_L_ immunostaining of minimal/mild endometriosis samples, for both epithelial (*P* = 0.153, gamma test) and stromal cells (*P* = 0.767, gamma test).

A negative and significant correlation was observed between OB-R_L_ transcripts and PF leptin/BMI ratio in ectopic endometrium (*R* = −0.49,  *P* = 0.019, Spearman's correlation). This was not observed in the eutopic endometrium of controls (*R* = 0.06,  *P* = 0.8). 

## 4. Discussion

In the present study, we found a significantly higher serum leptin/BMI ratio in the endometriosis group, as well as a significantly higher expression of leptin and OB-R_L_ transcripts in the ectopic endometrium compared to the eutopic endometrium of patients with endometriosis and normal pelvis controls. These results suggest a putative role of leptin in the development of endometrial implants.

Despite the strong relationship between leptin and BMI, only a few studies have analyzed the leptin/BMI ratio instead of leptin concentrations in women with endometriosis [13, 32]. Using the leptin/BMI ratio allowed us to control the influence of individual body weight on leptin secretion, thus increasing the accuracy of results. 

In the present study, we found a trend toward higher levels PF leptin/BMI ratio in the presence of endometriosis. Some investigators have also reported higher PF leptin in women with endometriosis as compared to controls [2, 6, 9, 10, 12]. Concerning the severity of endometriosis, whereas this aspect was not correlated with serum and PF leptin/BMI ratio in the present study, it was inversely correlated with PF leptin levels in the study by Mahutte et al. [8]. Wertel et al. [13], Bedaiwy et al. [5], and Gungor et al. [7] found a positive correlation between endometriosis severity and leptin levels, while Barcz et al. [11] did not observe any correlation. Such discrepancies are not surprising, given the fact that these studies differ widely with regard to patient characteristics (including age, BMI, and endometriosis severity), endpoints, and stratification (or not) of the primary endometriotic lesion by anatomical location. Nevertheless, despite the specificities of each study, all seem to indicate (at least with the current commercially available kits) that PF leptin alone is not a good marker to screen for the presence, location, or severity of endometriosis. In fact, recent studies have shown that combining the serum concentration of various proteins that are differentially expressed in women with and without endometriosis, including leptin, would greatly increase diagnostic accuracy as compared to assaying each protein alone [33, 34].

Concerning the associations between leptin levels and gene expression and the menstrual cycle phases, our results are consistent with previous findings that showed no significant differences in leptin levels between follicular and luteal phases [5, 6]. Previous studies have shown higher leptin expression during the implantation period [16] and total and long-form leptin receptor gene expression throughout the menstrual cycle, with increased expression in the early luteal phase [3]. In the present study, laparoscopies were scheduled preferentially in the secretory phase in order to evaluate the association between leptin and endometrial differentiation, rather than proliferation. However, because some participants had their samples collected in the proliferative phase, we observed that serum and PF leptin/BMI ratios were comparable in the two phases in both endometriosis and control participants. In addition, the estradiol and progesterone levels recorded in each cycle phase (proliferative or secretory) were similar in endometriosis patients and controls. 

It should be noted that our control group did not include strictly normal women, but rather patients without pelvic disease at laparoscopic inspection. Some previous studies have investigated the association between leptin, infertility, and chronic pelvic pain. Wertel et al. [13] have shown that serum and peritoneal fluid concentrations of leptin were similar in fertile and infertile patients with endometriosis, as well as in patients with unexplained infertility and tubal ligation. Tao et al. [14] also found no difference in peritoneal fluid leptin levels of patients with endometriosis and infertility compared to a group with fallopian-associated infertility and controls with myoma. According to Barcz et al. [11], infertile patients had higher leptin levels than patients with chronic pelvic pain, regardless of the presence of endometriosis. However, fertility was not tested in all patients with chronic pelvic pain in that study. Bedaiwy et al. [5] observed higher peritoneal fluid leptin levels in patients with endometriosis versus patients with unexplained infertility or those undergoing laparoscopy for tubal ligation or reversal of tubal ligation. There was no difference in leptin levels between the unexplained infertility and tubal ligation/reversal of tubal ligation groups. In a subgroup of endometriosis patients presenting pelvic pain, a positive correlation was found between peritoneal fluid leptin concentration and severity of symptoms, except when infertility was the main presenting symptom. Thus, the presence of infertility associated with endometriosis does not seem to influence leptin concentrations, but leptin might play a role in thepathophysiology of pain associated with endometriosis. It is important to note that, in our study, 68% of patients underwent laparoscopy for tubal ligation. These patients had a laparoscopically normal pelvis and were symptomfree. Five patients complained of infertility, and only two reported pelvic pain. All suspicious lesions were evaluated by histopathology. Thus, our control group may be regarded as adequate for the purpose of this study. 

We observed that expression of leptin and OB-R_L_ transcripts was significantly higher in ectopic versus eutopic endometrium of patients with endometriosis and normal pelvis controls. In addition, we detected leptin mRNA in almost all eutopic and ectopic endometrium samples, a finding that might be attributable, at least in part, to the method used in our study, as real-time RT-PCR is more sensitive to smaller amounts of mRNA. 

Our choice to analyze only the long form of the leptin receptor was based on the evidence that this isoform has the highest transcriptional activity [35]. Lima-Couy et al. [16] assessed the total, long, and short isoforms of leptin receptor in eutopic endometrium of patients with moderate and severe endometriosis and observed an increase in receptor expression during the period of embryo implantation, with no differences between eutopic endometrium from patients with endometriosis and controls. 

Other authors have identified leptin and OB-R transcripts in eutopic endometrium. Kitawaki et al. [3] identified the long form of the receptor in 84% of endometrial samples versus 85% in our study, including eutopic endometria of patients and controls. In an in vitro study, González et al. [36] demonstrated that the presence of leptin and leptin receptor mRNA in endometrial epithelial cells and embryos could be related to the embryo implantation process. Kitawaki et al. [3] also showed fluctuations in the expression of OB-R in endometrium, with a peak in the early secretory phase.

OB-R_L_ mRNA expression was negatively correlated with PF leptin/BMI ratio in ectopic endometrium. This observation is in agreement with a previous study, which found that treatment of stromal cell cultures with leptin reduces OB-R_L_ [17], suggesting modulation between leptin and its receptor in endometriosis. Later, the same authors demonstrated that, under hypoxic conditions, leptin gene expression was increased in both eutopic and ectopic endometrial stromal cells and that this process is likely to be mediated directly by hypoxia-inducible factor-1*α* (HIF-1*α*). Considering that leptin may promote cell proliferation, a low-oxygen environment in endometriotic implants could lead to stimulation of leptin gene expression, increasing the proliferation of endometrial stromal cells, with subsequent implantation of these cells in the peritoneum [18]. Our finding of a significant negative correlation between OB-R_L_ mRNA expression and peritoneal fluid leptin/BMI ratio in endometriosis also suggests that peritoneal fluid leptin/BMI ratio might have greater influence on the molecular regulation of OB-R_L_ receptor than the circulating leptin/BMI ratio. However, the experimental design of the present study does not allow us to confirm this possibility. Further *in vitro* studies are needed in order to determine the effect of different leptin concentrations on OB-R_L_ gene expression in isolated endometriotic cells.

Leptin is thought to play a role in endometriosis through its inflammatory and angiogenic properties. Using a rat model of endometriosis, Styer et al. [37] demonstrated that disruption of leptin signaling by administration of the pegylated leptin peptide receptor antagonist (LPrA) or nonfunctional leptin receptor (Lepr^dB^) inhibits the establishment and development of endometriosis-like lesions that resemble peritoneal endometriotic foci. The administration of recombinant VEFG to these animals led to an increase in the formation of endometrial glands, however, at a lower density in relation to controls. Therefore, leptin signaling seems to be a necessary component of lesion proliferation, initial vascular recruitment, and maintenance of neoangiogenesis in a murine model of endometriosis. Recently, Oh et al. [19] showed in an *in vitro* model of cell culture from endometrioma and endometrial tissues of women without endometriosis that the expression of leptin receptor was significantly higher in endometriotic epithelial cells than in epithelial and stromal cells of the normal endometrium or in endometriotic stromal cells. In addition, leptin treatment stimulated the proliferation of only endometriotic epithelial cells. In contrast, inhibition of JAK2/STAT3 and ERK signaling pathways of the leptin receptor induced a blockage of growth in these endometriotic epithelial cells through leptin stimulation. The increase in leptin and leptin receptor expression in the ectopic endometrium of women with endometriosis may lead to increased leptin signaling in implants, resulting in proliferation, neoangiogenesis, and maintenance of ectopic endometrial tissue [37]. 

The higher serum leptin/BMI ratio observed in the endometriosis group as compared to the control group may be due to endometriotic implants rather than adipose tissue, since leptin and OB-R_L_ transcripts were similar in the fat tissue of endometriosis and control participants. 

We were unable to confirm previous findings reporting more elevated PF leptin in milder disease [2, 6, 8]. However, the biological activity of the disease might be related to type rather than extent of lesion. In this sense, Gazvani et al. [38] showed that red lesions are more biologically active than white or black lesions. Further studies are needed to specifically study the relationships between leptin and its receptor transcripts according to the type of lesions found at laparoscopy.

One limitation of our study concerns the purity of cell populations in the ectopic endometrium samples. Tissues obtained from lesions contain a mixture of cell types, including leukocytes and peritoneal fibroblasts, in addition to ectopic endometrium [39]. Thus, there is a small risk that peritoneal cells may account for some of the results observed.

## 5. Conclusions

The present data suggest that serum leptin/BMI ratio is associated with the presence of endometriosis. Nevertheless, the clinical applicability of the leptin/BMI ratio for prediction of endometriosis still requires confirmation. Moreover, the increased expression of leptin and OB-R_L_ in ectopic endometrium suggests a modulatory interaction between leptin and its active receptor and a role of leptin, an inflammatory and angiogenic cytokine, in the initiation or development of endometrial implants.

## Figures and Tables

**Table 1:** Characteristics of women with endometriosis and normal pelvis controls.

	Endometriosis	Controls
*N*	28	17
Age (years)^a^	32 ± 7	33 ± 5
BMI (kg/m²)^a^	25.6 ± 4.5	25.2 ± 3.5
Estradiol (pg/mL)		
Proliferative	122 (37–160)	64 (18–144)
	(*n* = 5)	(*n* = 4)
Secretory	104 (56–226)	91 (55–132)
	(*n* = 23)	(*n* = 13)
Progesterone (ng/mL)		
Proliferative	0.2 (0.18–0.79)	0.42 (0.21–0.86)
	(*n* = 5)	(*n* = 4)
Secretory	3.6 (0.56–11.25)	4.15 (0.59–9.1)
	(*n* = 23)	(*n* = 13)

^a^Age and BMI are expressed as mean ± SD.

No significant difference in age, BMI, estradiol, and progesterone between endometriosis and control groups (Student's *t*-test).

BMI: body mass index.

**Table 2:** Serum and peritoneal fluid leptin/BMI ratio according to stage of endometriosis.

	Controls	Endometriosis	*P*	rASRM		*P* ^a^
				Stage I/II	Stage III/IV
	0.41	0.61		0.56	0.78	
Serum leptin/BMI	(0.22–0.71)	(0.41–0.95)	0.04	(0.28–0.99)	(0.43–0.96)	0.08
	*n* = 17	*n* = 28		*n* = 13	*n* = 15	
Peritoneal fluid	0.44	0.7		0.54	0.71	
leptin/BMI	(0.28–0.73)	(0.45–1.18)	0.07	(0.28–1.36)	(0.59–1.15)	0.12
	*n* = 17	*n* = 23		*n* = 12	*n* = 11	

Data are presented as median and interquartile range (Mann-Whitney).

^a^Controls versus rASRM stage I/II versus rASRM stage III/IV (Kruskal-Wallis).

rASRM stages: revised American Society for Reproductive Medicine classification (15).
